# Alteration of adipose tissue immune cell milieu towards the suppression of inflammation in high fat diet fed mice by flaxseed oil supplementation

**DOI:** 10.1371/journal.pone.0223070

**Published:** 2019-10-17

**Authors:** Samina Bashir, Yadhu Sharma, Deeba Jairajpuri, Faraz Rashid, Md. Nematullah, Farah Khan

**Affiliations:** 1 Department of Biochemistry, Jamia Hamdard, New Delhi, India; 2 Department of Medical Biochemistry, College of Medicine and Medical Sciences, Arabian Gulf University, Manama, Bahrain; 3 121 DHR, Udyog Vihar, Phase IV, Gurugram, Haryana, India; Universidade do Estado do Rio de Janeiro, BRAZIL

## Abstract

The present study evaluates the effect of flaxseed oil (FXO) supplementation on adipose tissue macrophages (ATM’s), E and D series resolvin (Rv) levels and adipose tissue inflammation. Male C57BL/6J mice were divided into five groups (n = 5): lean group (given standard chow diet), HFD group given high fat diet (approx. 18 weeks) till they developed insulin resistance and 4, 8 or 16 mg/kg group (HFD group later orally supplemented with 4, 8 or 16 mg/kg body weight flaxseed oil) for 4 weeks.The present study showed that FXO supplementation led to enhanced DHA, EPA, RvE1-E2, RvD2, RvD5- D6, IL-4, IL-10 and arginase 1 levels in ATMs together with altered immune cell infiltration and reduced NF-κB expression. The FXO supplementation suppresses immune cell infiltration into adipose tissue and alters adipose tissue macrophage phenotype towards the anti-inflammatory state via enhancement of E and D series resolvins, arginase 1 expression and anti-inflammatory cytokines level (IL-4 and IL-10.) leading to amelioration of insulin resistance in flaxseed oil supplemented HFD mice.

## Introduction

Obesity is a state of chronic low-grade inflammation that originates due to the altered resolution of inflammation. This inflammatory condition disturbs the balance between the metabolic pathway and immune system leading to obesity-associated conditions such as insulin resistance, type 2 diabetes, atherosclerosis and non-alcoholic fatty liver disease [1].Adipose tissue-resident immune cells, especially macrophages are the key contributors to initiate inflammatory cascade in adipose tissue. The infiltration of macrophages into the adipose tissue and their polarization towards pro-inflammatory nature has evidently been linked to the onset of obesity-associated complications in humans as well as in rodent models [2]. The process of macrophage polarization involves switching of adipose tissue macrophages (ATMs) which perform homeostatic functions via IL-4, IL-10, arginase 1 etc. and are called as M2 macrophages (anti-inflammatory) towards M1 macrophages (pro-inflammatory) that secrete various pro-inflammatory mediators (TNF-α, IFN-ϒ and IL-6) maintain inflammatory milieu [3].

The altered resolution of inflammation under conditions of obesity and insulin resistance may be modulated by therapeutic interventions or modification in diet/ lifestyle that lead to macrophage switching [4]. On exposure to saturated fatty acids, macrophages in vitro express increased pro-inflammatory genes and cytokines (e.g., TNF-α, IL6, and CXCL1/KC) while supplementation with n-3 fatty acids from dietary sources like fish oil leads to reduced pro-inflammatory cytokines production and inhibition of M1 states in ATMs [4, 5]. More importantly, a group of pro-resolving lipid mediators that include resolvins have been delineated for resolution of acute inflammation and enhance phagocytosis by macrophages [6]. Eicosapentaenoic acid (EPA) and docosahexenoic acid (DHA) are enzymatically converted to E-series resolvins (RvE1-2) and D-series resolvins (RvD1-6), respectively, and are considered as highly beneficial bioactive mediators to combat obesity-associated inflammation. D series resolvins have been found to be important in protecting the host from obesity-induced insulin resistance and hepatic steatosis in murine models in vivo [7]. RvE1 and RvE2 act as endogenous receptor antagonists for the leukotriene B4 receptor BLT-1, making them potent regulator of neutrophil trafficking to sites of inflammation [8].Despite extensive investigations on the effect of dietary supplementationon adipose tissue physiology, their therapeutic/pharmacological potential remains elusive. Moreover, how the balance of lipid mediators changes during the course of obese and insulin resistance is still unclear.

Flaxseed oil or *Linumusitatissimum* L., is a rich source of alpha-linolenic acid (ALA), a long chain n-3 fatty acid which is converted by the body into EPA and DHA [9].These fatty acids are known ligands for peroxisome proliferator-activated receptors (PPARs) especially PPAR-α which are regulators of cell metabolism. The activity of liver enzymes D6 and D5 fatty acid desaturases that are involved in biosynthesis of EPA and DHA from n-3 precursor ALA is affected by insulin resistance, hypercholesterolemia and non-alcoholic fatty liver disease [10]. The decrease in the biosynthesis of EPA and DHA in conditions such as diet induced obesity leads to disturbance in cell metabolism via affecting fatty acid oxidation, antioxidant responses etc. Therefore, supplementation of oils rich in ALA or EPA/DHA is required to cover the minimum nutritional requirements [11].

In the present study, we investigated the therapeutic effect of flaxseed oil supplementation on the obese insulin-resistant model. We observed that FXO supplementation to high-fat diet fed obese insulin-resistant mice increases EPA, DHA, resolvin RvE1, RvE2, RvD2, RvD6 levels, inhibits macrophage infiltration into the adipose tissue and reduces pro-inflammatory cytokines production by ATMs leading to improved insulin sensitivity through adipose tissue remodelling.

## Materials and Methods

### 2.1 Materials

LC grade solvents (Biosolve, Dieuze, France), Cleanert S, C18 solid-phase extraction columns (500 mg/ 3ml), Waters ACQUITY UPLC BEH- C18 column (2.1 mm X 50mm, 1.7μm); deuterated (d) internal standards 17(S)-Resolvin D1-d5_,_EPA, DHA was purchased from Cayman Chemical (Ann Arbor, MI). All the ELISA kits for cytokine analysis were purchased from e-Bioscience, USA. Mouse insulin ELISA kit was obtained from Mercer EXPert, USA. Collagenase II, anti-arginase 1, anti-β-actin and anti-NFκB/P65 antibodies were purchased from Sigma-Aldrich Co. (USA). Light Cycler^®^ 480 SYBR Green I Master kit was purchased from Roche, USA.RNA isolation kit was from MP Biomedicals, USA. Membrane filters (100 μm) were purchased from Millipore (USA). All other chemicals were of analytical grade and procured from standard commercial sources in India.

### 2.2 Animal model

Male C57BL/6J mice (6 weeks old, 20-25g) were approved by the University’s animal ethics committee [173/Committee for the Purpose of Control and Supervision of Experiments on Animals (CPCSEA), Government of India]. Mice were randomly divided into five groups (n = 5):control group received standard rodent diet, HFD group was fed high-fat diet (60% calories derived from fat, 20% from carbohydrate, and 20% from protein) and other HFD groups were orally administrated with flaxseed oil (FXO4, 8 or 16 mg/kg body weight). The FXO dosage was calculated using the formula: Human effective dose (mg/kg) = mice dose (mg/kg)×mice Km\human Km (Human Km = 37, mouse Km = 3) [12]. Therefore, the dosage of 4, 8, 16 mg/kg BW in mice corresponds to 0.32, 0.64, 1.29 mg/kg BW in humans. The dietary regimen (standard rodent diet &HFD) lasted for a period of 18 weeks and flaxseed oil supplementation was given for a period of 4 weeks. The high-fat diet was prepared as described earlier and the total calories (per gram) from the diet were measured using bomb calorimeter (IVRI, India)[13]. At the end of the study, mice were fasted overnight and orally gavaged with dextrose (1.5 g/kg BW) [14]. The blood (250μl) was collected at 0, 15, 30, and 60 minutes after the dextrose load for the measurement of blood glucose and insulin levels by glucose oxidase method and mouse ELISA kit (Mercer EXPert, USA) respectively. Blood triglyceride and cholesterol levels were measured as per the protocol mentioned inrespective assay kits (SPAN diagnostics kit, Surat, India). Mice were sacrificed by giving overdose of ketamine/xylazine and epididymal, inguinal, retroperitoneal and mesenteric fat pads were dissected to measure fat pad weight. Adiposity index was calculated as sum of the fat pads/(bodyweight—fat padweight) X 100[15]. The FXO dosage found most efficient in ameliorating metabolic parameters was used for further study. The epididymal fat pad was referred as adipose tissue for all the histological and immunological experiments performed.

### 2.3 Adipose tissue histology and NF-κB immunohistochemistry

6 μm thick paraffin-embedded adipose tissue sections were stained with hematoxylin and eosin for histopathological examination. For immunohistochemical analysis of NF-κB expression, paraffin-embedded adipose tissue sections were stained with the NF-κB monoclonal antibody (Sigma, USA) at 1:200 dilution, overnight at 4°C. The slides were washed three times in TBS and incubated for 2 h at room temperature with horseradish peroxidase (HRP) conjugated secondary antibody (Sigma, USA). The immunostained sections were examined for diffuseness of the staining and graded as 0, no staining; 1, staining of 25%; 2, staining between 25% and 50%; 3, staining between 50% and 75% and 4, staining > 75%. All images were observed at 100X magnification.

### 2.4 Cytokine analysis and arginase 1 expression

Macrophages were isolated from the epididymal fat pad of mice as described earlier using collagenase II method [16]. Briefly, epididymal adipose tissue was isolated, finely chopped, incubated with collagenase II and the cell suspension obtained was filtered through a100-μm membrane filter and centrifuged. The pellet containing stromal vascular fraction was plated to detect adherent cells (ATM’s). The obtained adherent cells were washed with PBS and used for further analysis. For cytokine analysis, ATMs were isolated and then cultured in 5% CO_2_ at 37°C for 72 h. After incubation, the supernatant was collected from the cultured cells and filtered by a 0.22μm filter. Collected supernatant was used for cytokines analysis (TNF-α, TGF-β, IL-1β, IL-2, IFN-γ, IL-10 and IL-4) according to manufacturer’s instructions (e-Bioscience, USA).

To check the arginase 1 mRNA levels, total RNA was isolated from ATMs using RNA isolation kit according to the instructions of the manufacturer (MP Biochemicals, USA). 200 ng total RNA was reverse transcribed and arginase 1mRNA levels were analyzed using Light Cycler^®^ 480 SYBR Green I Master kit (Roche, USA). The following primer pairs were used:Arginase1F:5’AAGAAAAGGCCGATTCACCT3’; R:5’CACCTCCTCTGCTGTCTTCC3’ and β-actin F: 5’TACAGCTTCACCACCACAGC 3’; R: 5’AAGGAAGGCTGGAAAAGAGC3’and fold change was normalized to b-actin expression and calculated using the 2^-ΔΔCT^ calculation method [17].

ATMs were homogenized in RIPA buffer containing protein inhibitor cocktail. The protein sample obtained was separated by 10%S.D.S-PAGE, and then blotted to 0.22 μm PVDF membranes (Millipore, USA). The blotted membranes were first blocked with skimmed milk, then washed with Tween-20/PBS, and subsequently incubated with anti- arginase 1 antibody or anti-β-actin antibody (Sigma, USA). After the incubation period, membranes were incubated with species-specific HRP-conjugated secondary antibody. HRP activity was detected by ECL reagent (Millipore, USA) and bands were analyzed by ImageJ software on a densitometer.

### 2.5 Analysis of E and D series resolvins levels in adipose tissue macrophages by LC-MS

For LC-MS analysis, internal labeled standards 17(S)-Resolvin D1-d5 (1 ng) in 2 ml of ice-cold methanol was added to each sample to facilitate quantification and sample recovery. Each sample was gently sonicated and kept on ice for 1h to allow protein precipitation and then centrifuged at 1,200 g at 4°C for10 min. Supernatants were collected and brought to less than 1 ml of methanol content in a gentle stream of nitrogen gas onto an automated evaporation system (miVac Concentrator, ENGLAND). C18 (Cleanert) cartridges were placed into negative pressure SPE system (Vacuum Manifold, Millipore) and were equilibrated with 2ml of methanol and 5 ml of H_2_O. 9 ml of H_2_O (pH 3.5, HCl) was then added to the samples, and the acidified solutions were rapidly loaded onto the conditioned C18 columns that were washed with 4 ml of H_2_O to neutralize the acid. 5 ml of hexane was then added and products eluted with 9 ml of methyl formate. Products were brought to dryness using the automated evaporation system (miVac Concentrator, ENGLAND) and immediately suspended in water-methanol (50:50 v/v) for LCMS- MS automated injections. The LCMS-MS system, a Shimadzu LC-30AD HPLC and a Shimadzu SIL-30AC autoinjector (Shimadzu, Kyoto, Japan), paired with a QTrap 6500+ (SCIEX, Framingham, MA), was routinely employed. Waters ACQUITY UPLC BEH- C18 column (2.1 mm X 50 mm, 1.7μm) was kept in a column oven maintained at 50°C and targeted lipids were eluted with a mobile phase consisting of water-methanol-acetic acid of 45:55:0.01 (v/v) initially equilibrated for 2 mins, that was ramped to 15:85:0.01 (over 8 min and then to 2:98:0.01 for the next 2 min). This was subsequently maintained at 2:98:0.01 for 2 min, and the flow rate was maintained at 0.6 ml/min. The QTrap 6500+ was operated in negative ionization mode using scheduled multiple reaction monitoring (MRM) coupled with the information-dependent acquisition (IDA) and an enhanced product ion scan (EPI). The scheduled MRM window was 100 ms, and each targeted lipid mediator compound parameter was optimized individually as described earlier [18]. Linear calibration curves for combined EPA and DHA standard were obtained with r2 values in the range 0.99.

### 2.6 Statistical analysis

Data were statistically analyzed using one way ANOVA followed by Tukey post test using GraphPad Prism (ver 7) software to determine significant differences in the data of various groups. p-value less than 0.05 were considered statistically significant. All values are expressed as a mean ± standard deviation. For glucose tolerance test and insulin tolerance test graphs were plotted as 95% confidence limit and analyzed by two-way ANOVA followed by Tukeymultiple comparison tests.

## Results

### 3.1 Effect of FXO supplementation on metabolic parameters

**Fig 1:**
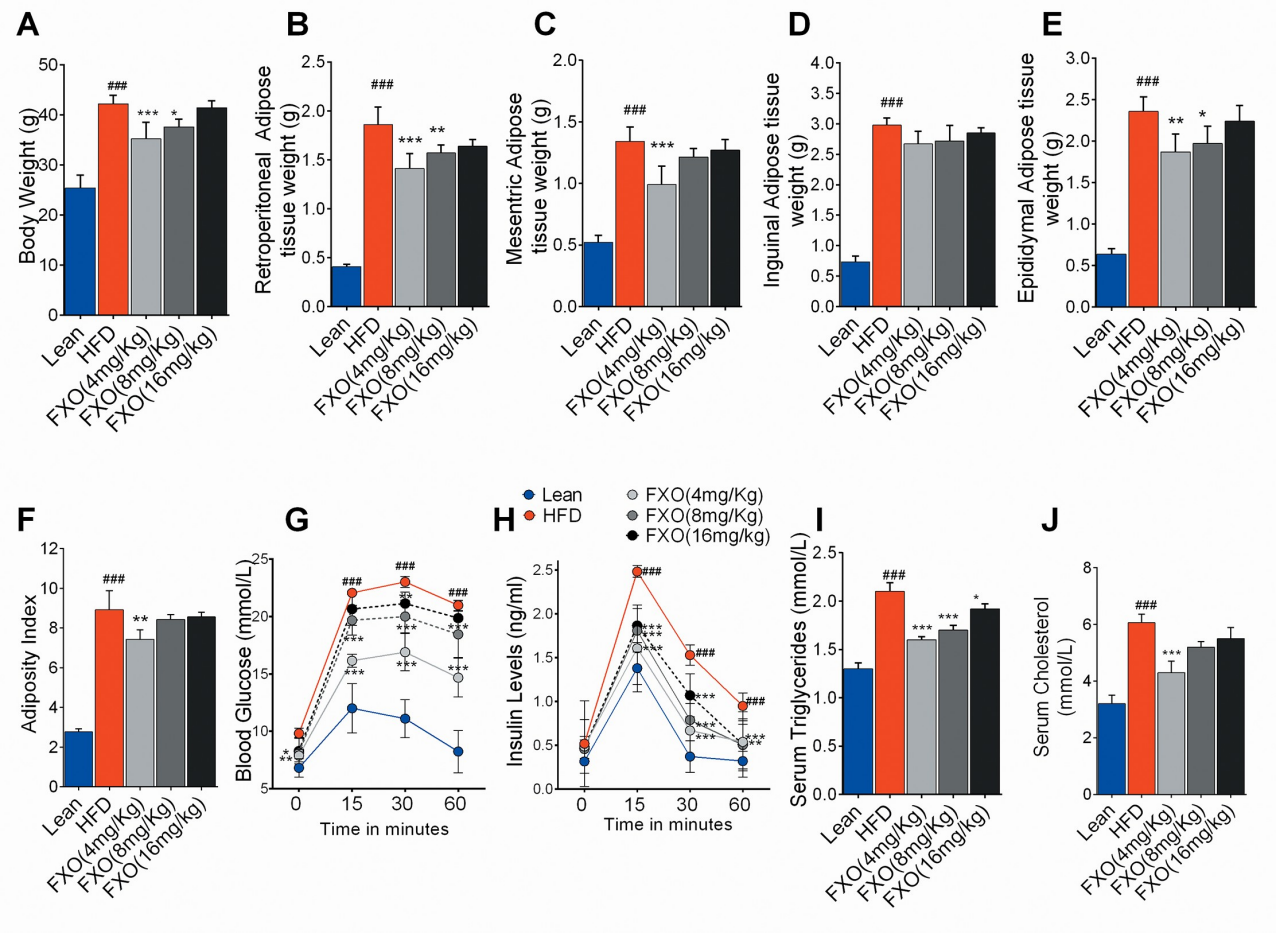
Effect of FXO on metabolic parameters. (A) Body weight (B-E) Adipose tissue weight (retroperitoneal, mesenteric, epididymal and inguinal) (F) Adiposity index (G) OGTT (H)Blood insulin (I) Serum triglyceride (J)Serum cholesterol. Mice underwent an OGTT (1.5 g/kg BW glucose gavage) after FXO supplementation. Statistically significant values are denoted by #p<0.05, ##p<0.01, ###p<0.001 from one way or two way ANOVA followed by Tukey post testwhenHFD is compared with lean group and *p < 0.05, **p <0.01,***p <0.001 when FXO supplemented groups are compared with HFD. Values presented as mean ± S.D., n = 5.

The development of obese insulin-resistant model was evaluated by measuring metabolic parameters. As anticipated, HFD group showed significant 41% increase in body weight in comparison to lean group which is significantly reduced in FXO 4mg/kg BWgroup (p<0.001) and 8 mg/kg BW (p<0.05) groups (Fig 1A). However, the higher dosage of FXO supplementation i.e. 16mg/kg BW does not significantly reduce the body weight. The retroperitoneal, mesenteric, inguinal and epididymal fat pad weights from mice were measured in all the groups (Fig 1B–1E). A significant decrease was observed in the epididymal and retroperitoneal fat pad weight from FXO 4mg/kg BWand 8 mg/kg BW dosage groups in comparison to HFD. In comparison to HFD group, FXO 4mg/kg BW supplementation reduced weight from 1.86±0.18 g to 1.41±0.15 g, 1.34±0.12 g to 0.99±0.15 g, 2.98±0.12 g to 2.67±0.21 g and 2.36±0.18g to 1.87±0.22g in retroperitoneal, mesenteric, inguinal and epidydimal fat pads, respectively. However, an insignificant decrease was observed in inguinal fat pads weight in all the dosage groups when compared with HFD. Also, 4 mg/kg body weight group showed a significant (p<0.01) decrease in adiposity index in comparison to HFD group (Fig 1F).

Oral glucose tolerance test (OGTT) was carried out in all the groups at 0, 15, 30 and 60 minutes post 1.5 g/kg BW dextrose oral gavage in mice fasted for 6 h prior to the test. All the groups (FXO4, 8 and 16 mg/kg BW dosage groups) showed a significant decrease in serum glucose level in comparison to HFD mice and also showed reduced serum levels of insulin 60 minutes after oral gavage with glucose (Fig 1G). Dosage group of FXO4mg/kg BW showed most significant (p < 0.001) effects on the reversal of insulin resistance (Fig 1H).To analyze the impact of FXO supplementation on lipid metabolism, serum triglyceride and cholesterol levels were measured. Mice fed a high-fat diet had higher triglyceride and cholesterol content in serum (2.1 ± 0.09 mmol/L and 6.0±0.3 mmol/L, respectively) than lean mice (1.3 ±0.06 mmol/L and 3.2± 0.3 mmol/L, respectively) (Fig 1I & 1J). FXO 4mg/kg BW supplementation significantly reduced (p<0.001) serum cholesterol levels when compared to HFD group. Flaxseed oil had a significant effect on lowering serum triglyceride levels at all FXO dosage in comparison to HFD mice.

As FXO 4mg/kg BWsupplementation was most efficient in improving metabolic health of high-fat diet fed mice among all the dosage groups, FXO 4mg/kg BW dosage was used for further study along with the lean and HFD group.

### 3.2 Effect of FXO supplementation on adipose tissue inflammation

**Fig 2:**
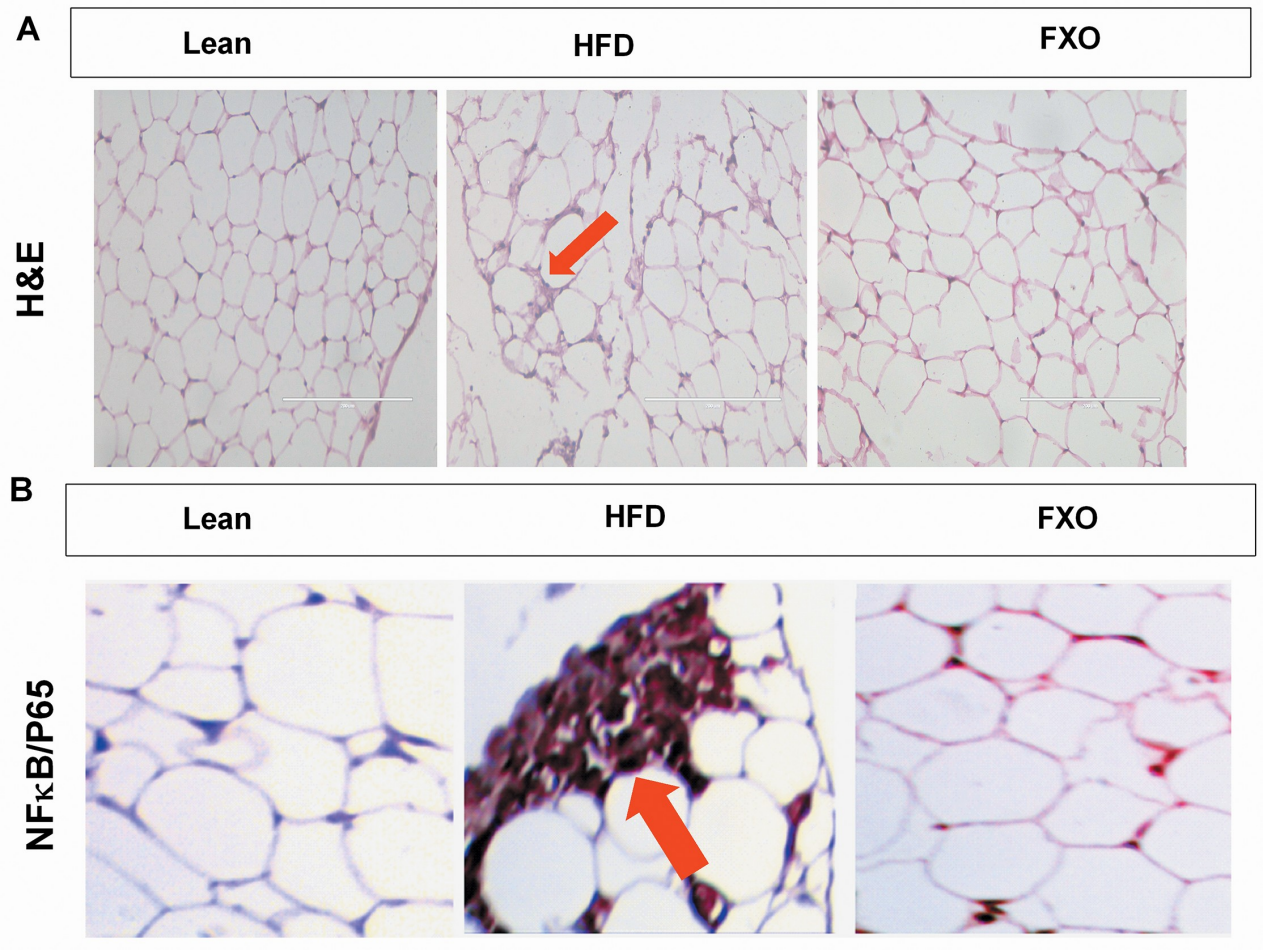
**Effect of FXO supplementation on adipose tissue inflammation (A) H& E stained sections of adipose tissue.** The infiltrated immune cells into the adipose tissue are marked by red Arrow. Scale bar = 200 μm. (B) Representative immunohistochemistry of NF-κB in adipose tissue. Score 1 = 25% staining in lean mice, score 4 = >75% in HFD mice and score 2 = 25–50% staining in 4 mg/kg BW group. Arrows indicate the staining areas. 100X magnification.

Histopathological analysis of epidydimal adipose tissue using hematoxylin and eosin dyes showed a massive infiltration of immune cells in HFD group (Fig 2A). FXO 4mg/kg BW supplementation was observed to cause a reduction in this infiltration. While analyzing obesity-associated inflammation, the analysis of NF-κB expression is of paramount importance as this transcription factor drives expression of various pro-inflammatory mediators. To analyze the effect of FXO supplementation on NF-κB expression, immunohistochemistry was done using anti-NF-κB/p65 antibody. The epididymal fat pad showed increased levels of NF-κβ in the HFD mice on immunohistochemical analysis as shown in Fig 2B. Supplementation with FXO resulted in a reduction in NF-κβ staining in comparison to HFD mice. Very low expression of NF-κβ was observed in lean group (Score 1) and moderate diffusion of staining was observed in 4mg/kg BW group (Score 2) in comparison to the HFD mice (Score 4) which showed enormous diffused staining in the tissue.

### 3.3 Effect of FXO supplementation on cytokine profile and arginase 1 expression

**Fig 3:**
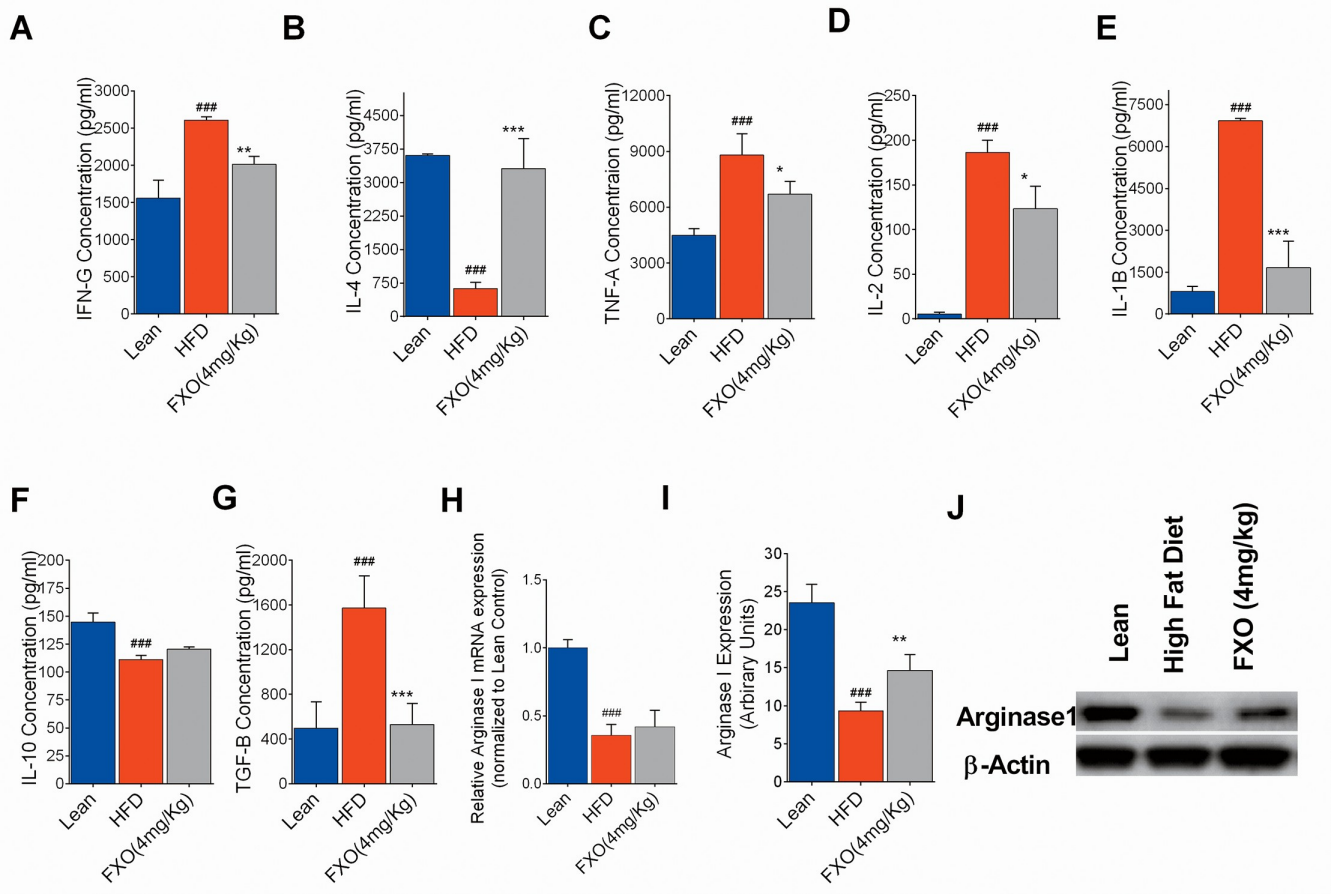
**Effect of FXO supplementation on cytokines and arginase 1 expression(A-G).** Cytokines IFN-γ, IL-4, TNF-α, IL-2, IL-1β, IL-10 and TGF-β level analyzed from adipose tissue macrophages in lean, HFD and FXO 4 mg/Kg BW group. Data represented as mean±S.D. (n = 3).Statistically Significant values are denoted by ###p<0.001 from one way ANOVA followed by Tukey post test when HFD is compared with lean group and *p < 0.05, **p < 0.01, ***p < 0.001 when FXO supplemented group is compared with HFD. (H) Graphical representation of fold changes in arginase 1 mRNA levels of adipose tissue macrophages in comparison to β-actin mRNA (constitutive gene) done by real-time PCR. Data represented as mean±S.D. (n = 3). (I &J) Western blot and graphical representation of % increase in arginase 1 expression calculated by ImageJ software. Data are presented as mean ± S.D. (n = 2). Statistically Significant values are denoted by ###p<0.001 from one way ANOVA followed by Tukey post test when HFD is compared with the lean group and **p < 0.01 when FXO supplemented group is compared with HFD.

Further, we checked the cytokine levels secreted by ATMs to throw some light on the phenotype of macrophages present predominantly. The effect of FXO supplementation on cytokine profile of ATMs was analyzed. HFD group displayed significantly high levels (p<0.001) of pro-inflammatory cytokines (TNF-α, IFN-γ, IL-1β, and IL-2) and diminished anti-inflammatory cytokine levels (IL-4 and IL-10) in comparison to lean group (Fig 3A–3F). FXO supplementation was observed to cause a significant shift in the cytokine profile from pro-inflammatory to the anti-inflammatory one. Reportedly, TNF-α contributes to the increased TGF-β expression during obesity. We also observed significantly elevated TGF-β expression in HFD group in comparison to lean group which was observed to be suppressed by FXO supplementation (Fig 3G).

Arginase 1 is a marker for the anti-inflammatory macrophages, hydrolyzing arginine to urea and ornithine, a precursor of various polyamines involved in the resolution of inflammation via repair mechanisms [19]. It also inhibits nitric oxide-induced inflammation by competing iNOS for the same substrate i.e. arginine. Therefore, changes in arginase 1 expression in adipose tissue macrophages have an enormous impact on obesity-associated adipose tissue inflammation. Analysis of relative expression of arginase1 mRNA compared to housekeeping gene β- actin was carried out by RT-PCR (Fig 3H). The results obtained suggest that high-fat diet causes almost three-fold reduction in arginase 1 mRNA levels in adipose tissue macrophages as compared to the lean group. FXO supplementation caused an insignificant upregulation in arginase 1 mRNA expression when compared to HFD mice. Further, the arginase 1 expression was evaluated at the protein level (Fig 3I & 3J). The levels of arginase 1 in HFD group were observed to be significantly two-fold reduced than in lean group which was significantly increased (p<0.05) in FXO supplemented group.

### 3.4 Effect of FXO supplementation on levels of E and D series Resolvins

**Fig 4:**
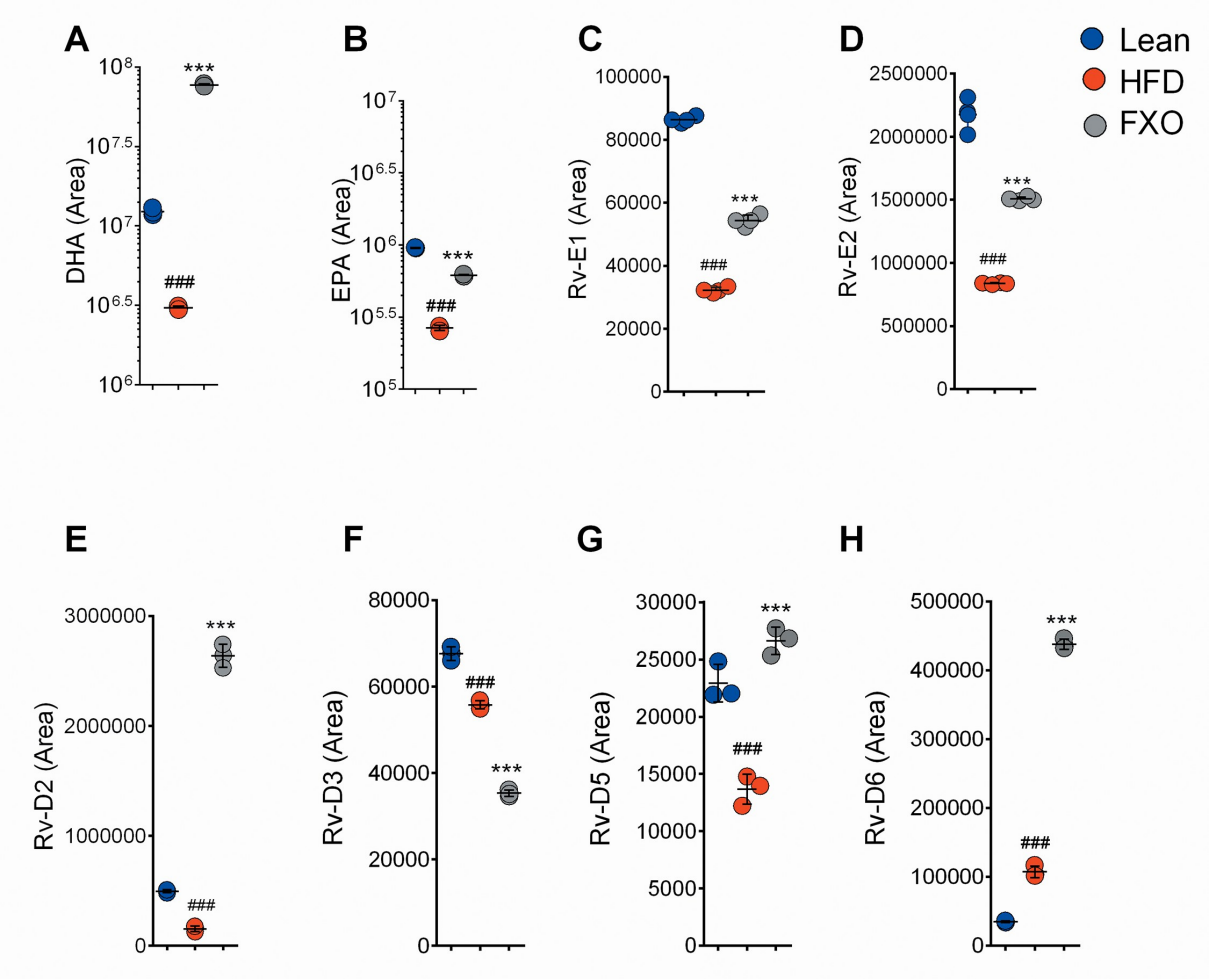
**Effect of FXO supplementation on (A) DHA (B) EPA (C) RvE1 (D) RvE2 (E) RvD2 (F) RvD3 (G) RvD5 and (H) RvD6 levels in adipose tissue macrophages.** Values presented as mean ± S.D., n = 3. Statistically Significant values are denoted by ###p<0.001 from one way ANOVA followed by Tukey post test when HFD is compared with the lean group and ***p < 0.001 when FXO supplemented group is compared with HFD.

FXO is a rich source of EPA and DHA which are a precursor for the synthesis of E & D series resolvins, respectively [20]. To check the impact of FXO supplementation, the levels of E and D series resolvins (Rv-E and Rv-D, respectively) in ATMs were measured and represented as the peak area obtained by LC-MS. FXO4mg/kg BW supplementation significantly increased (p<0.001) the levels of EPA and DHA in comparison to HFD group thereby providing the raw material for the synthesis of resolvins in order to rectify macrophage-induced adipose tissue inflammation (Fig 4A & 4B). Resolvin E1 (Rv-E1) and resolvin E2 (Rv-E2) levels were significantly reduced (p<0.001) in HFD mice in comparison to lean group (Fig 4C & 4D). Consistent with the result of EPA levels, FXO supplementation significantly elevated the levels of Rv-E1 (68% increase) and Rv-E2 (80%) in comparison to HFD mice. Further, analysis of resolvin D2 (Rv-D2), D5 (Rv-D5) and D6 (Rv-D6) showed a significant increase (p<0.001) in 4mg/kg BW group when compared with lean as well as HFD group (Fig 4E, 4G and 4H). The reduced level of resolvin D3 (Rv-D3) has been observed in arthritic inflammation and increased levels were found to be associated with the late phase of resolution of inflammation. Surprisingly, in our study, the relative concentration of Rv-D3 was observed to be significantly reduced in HFD group in comparison to lean group which was further reduced by FXO supplementation (Fig 4F).

## Discussion

The development of obesity-related inflammation occurs when hypertrophic adipocytes secrete pro-inflammatory adipokines that cause macrophages to migrate into the adipose tissue. This leads to a shift in cytokine levels from anti-inflammatory nature to pro-inflammatory that further leads to systemic inflammation and insulin resistance [21]. Recently, Serhan and Colleagues identified and characterized novel resolving lipid mediators namely lipoxins, resolvins and protectins families of endogenously produced, lipid-derived substances that were found to play a dynamic role in the resolution of inflammation [22]. Among these lipid mediators, E-series resolvins (RvE1 and RvE2) derived from EPA and D-series resolvins (RvD1-D6) derived from DHA, play important role in the resolution of inflammation and aid in tissue remodeling [23].

As our previous study demonstrated the anti-inflammatory potential of FXO and its impact on insulin sensitization, the present work was designed to evaluate the effect of FXO supplementation on switching adipose tissue macrophages, via modulating resolvin levels. In agreement with previous reports, we found that FXO treatment improved insulin sensitivity in obese mice [24]. HFD mice treated with FXO 4mg/kg BW exhibited significant improvement in metabolic parameters such as body weight, fat pad weight, adiposity index, blood glucose levels, blood insulin levels, serum triglyceride and serum cholesterol levels when compared to another dosage groups that showed little impact on the same. Therefore, lean, HFD and FXO 4mg/kg BW group were used for further analysis. The lowest concentration 4mg/kg may be giving the most significant effects because although it’s a rich source of omega 6 fatty acids, higher concentrations of flaxseed oil adds to the positive energy that may exert additional burden on the metabolic machinery leading to insignificant or lower degree of resolution of adipose tissue inflammation. However, further studies are warrantedtounderstand theresult.

Adipose tissue macrophages are the major players in the development of obesity-associated adipose tissue inflammation, thus we carried out relative quantification of DHA, EPA, RvE1, RvE2, RvD2, RvD3, RvD5 and RvD6 levels in ATMs isolated from lean, HFD, and FXO 4mg/kg BW groups. The elevation in the levels of RvE1, RvE2, RvD2, RvD5 and RvD6 in FXO supplemented group along with their precursor viz. EPA and DHA in adipose tissue macrophages show the paradigm shift towards pro-resolving nature. The dietary alpha-linolenic acid (ALA) is the only source for the biosynthesis of EPA and DHA and these fatty acids are required for the formation of cell structures and signalling molecules which regulates cell metabolism by controlling gene expression [25]. The supplementation with dietary ALA might be compensating for the reduced activity of liver enzymes to synthesize EPA and DHA that in turn improves metabolism and in turn resolves adipose tissue inflammation. Previously, RvE1 has been demonstrated to improve various inflammatory disorders [26–28]. D series resolvins have been found to be important in protecting the host from obesity-induced insulin resistance and hepatic steatosis in murine models in vivo. Although a recent study has provided evidence that both RvD2 and RvD1can modulate the chronic inflammatory process that takes place in the adipose tissue of obese subjects, we could not detect the presence of RvD1 in adipose tissue macrophages [27]. Surprisingly, RvD3 levels were lowered in FXO supplemented group in comparison to HFD group and requires further study. N-3 fatty acids, EPA and DHA are known to be a precursor for the formation of anti-inflammatory, pro-resolving, and cytoprotective lipid mediators; our study is the first to report the changes in the levels of E and D series resolvins in adipose tissue macrophages [29].

Further, the histopathological analysis of H & E stained adipose tissue that showed markedly immune cells infiltrated adipose tissue from HFD mice in comparison to lean group and reduction in immune cell population upon FXO supplementation. Resolvins are known to be potentially involved in reversing inflammatory status through damping the production of pro-inflammatory cytokines (TNF-α and IFN-γ), modulation of inflammatory pathways and inhibition of chemokine formation [30]. In this study, we found that FXO treatment results in inhibition of pro-inflammatory cytokines (IL-2, TNF-α and IFN-γ) production and upregulation of anti-inflammatory cytokines (IL-4, IL-10).

It is reported that E-series resolvins inhibit the production of pro-inflammatory cytokines via modulating NF-κB pathway [31]. Accordingly, our results showed that FXO supplementation effectively downregulates the adipose tissue NF-κB expression. These results suggest that RvE1 and RvE2 may be involved in reducing production of pro-inflammatory cytokines through modulating NF-κB in FXO treated obese mice.

Earlier studies have shown that resolvins increase the level of arginase1 which is a known marker of anti-inflammatory macrophages [32]. Also, the D-series resolvin behaves like IL-4 cytokine that induces anti-inflammatory nature of macrophages. In the present study, a significant change in the level RvD2 level was observed. This finding suggests that RvD2 may be involved in switching ATMs toward anti-inflammatory state via regulating arginase 1 levels in FXO supplemented HFD mice. We did observe a significant downregulation of arginase 1 levels in HFD group when compared to the lean group.

In conclusion, our findings indicate that the flaxseed oil provides the raw material i.e. EPA and DHA for the synthesis of E and D series resolvins by adipose tissue macrophages and aid in adipose tissue remodeling via regulating inflammatory status (pro- or anti-) of adipose tissue macrophages thereby protecting against obesity-induced adipose tissue inflammation and insulin resistance. However, further studies are required to establish direct link between increased resolvin levels and resolution of adipose tissue inflammation.

## Supporting information

S1 Fig The chromatograms showing peak area for RvE and RvD samples that were normalized using the dv-RVD2 as internal standard before SPE extraction was conducted. (DOCX)

S2 Fig MRM based relative quantification of the EPA, DHA and each resolvin (A) Rv-E1(B) Rv-E2 (C) Rv-D2 (D) Rv-D3 (E) Rv-D5 (F) Rv-D6 (G) EPA (H) DHA.
(DOCX)

## Abbreviations

FXO: Flaxseed oil
HFD: High-fat diet
Rv: Resolvin
DHA: Docosahexaenoic acid
EPA: Eicosapentaenoic acid
NF-κB: Nuclear factor kappa-light-chain-enhancer of activated B cells
ATMs: Adipose tissue macrophages
LC-MS: Liquid chromatography-Mass spectroscopy
BW: Bodyweight
