# Supplementary material for: Alteration of adipose tissue immune cell milieu towards the suppression of inflammation in high fat diet fed mice by flaxseed oil supplementation
Source: PLoS One. 2019 Oct 17;14(10):e0223070. doi: 10.1371/journal.pone.0223070 (PMC6797118; doi:10.1371/journal.pone.0223070)
Supplement: S1 Fig — (DOCX) [file pone.0223070.s001.docx]

S1 Fig. The chromatograms showing peak area for RvE and RvD samples that were normalized using the dv-RVD2 as internal standard before SPE extraction was conducted.
