# Supplementary material for: Alteration of adipose tissue immune cell milieu towards the suppression of inflammation in high fat diet fed mice by flaxseed oil supplementation
Source: PLoS One. 2019 Oct 17;14(10):e0223070. doi: 10.1371/journal.pone.0223070 (PMC6797118; doi:10.1371/journal.pone.0223070)
Supplement: S2 Fig — MRM based relative quantification of the EPA, DHA and each resolvin (A) Rv-E1(B) Rv-E2 (C) Rv-D2 (D) Rv-D3 (E) Rv-D5 (F) Rv-D6 (G) EPA (H) DHA. (DOCX) [file pone.0223070.s002.docx]

S2 Fig. MRM based relative quantification of the EPA, DHA and each resolvin (A) Rv-E1(B) Rv-E2 (C) Rv-D2 (D) Rv-D3 (E) Rv-D5 (F) Rv-D6 (G) EPA (H) DHA. Data is represented with respect to relative area based quantification.

(A)

(B)

(C)

(D)

(E)

(F)

(G)

(H)
